# The effect of a mindfulness-based intervention in cognitive functions and psychological well-being applied as an early intervention in schizophrenia and high-risk mental state in a Chilean sample: study protocol for a randomized controlled trial

**DOI:** 10.1186/s13063-017-1967-7

**Published:** 2017-05-25

**Authors:** Álvaro I. Langer, Carlos Schmidt, Rocío Mayol, Marcela Díaz, Javiera Lecaros, Edwin Krogh, Aída Pardow, Carolina Vergara, Guillermo Vergara, Bernardita Pérez-Herrera, María José Villar, Alejandro Maturana, Pablo A. Gaspar

**Affiliations:** 1 Escuela de Psicología, Facultad de Medicina, Universidad Austral de Chile, Campus Isla Teja s/n., Valdivia, Chile; 2 Center for Interdisciplinary Studies on the Nervous System (CISNe), Universidad Austral de Chile, Valdivia, Chile; 3 Instituto Milenio para la Investigación en Depresión y Personalidad, Pontificia Universidad Católica de Chile, Macul, Santiago, Chile; 4 Clínica Psiquiátrica Universitaria, Hospital Clínico y Facultad de Medicina, Universidad de Chile, Recoleta, Santiago, RM Chile; 5 Biomedical Neuroscience Institute, Independencia, Santiago, Chile; 6 Escuela de Psicología, Universidad Adolfo Ibáñez, Diagonal Las Torres 2640, Santiago, Chile; 7 Instituto de Neurociencias Clínicas, Facultad de Medicina, Universidad Austral de Chile, Campus Isla Teja s/n., Valdivia, Chile; 8 Red de Salud Mental REDGESAM, Santiago, Chile; 9 Servicio de Psiquiatría Hospital del Pino, San Bernardo, Santiago, Chile; 10 Consulta Privada, Santiago, Chile; 11 Departamento de Neurociencias, Facultad de Medicina, Universidad de Chile, Santiago, Chile

**Keywords:** Mindfulness, Schizophrenia, High-risk mental state, Cognitive functions, Psychological well-being

## Abstract

**Background:**

According to the projections of the World Health Organization, 15% of all disabilities will be associated with mental illnesses by 2020. One of the mental disorders with the largest social impacts due to high personal and family costs is psychosis. Among the most effective psychological approaches to treat schizophrenia and other psychotic disorders at the world level is cognitive behavioral therapy. Recently, cognitive behavioral therapy has introduced several tools and strategies that promote psychological processes based on acceptance and mindfulness. A large number of studies support the effectiveness of mindfulness in dealing with various mental health problems, including psychosis. This study is aimed at determining the efficiency of a mindfulness-based program in increasing cognitive function and psychological well-being in patients with a first episode of schizophrenia and a high risk mental state (those at risk of developing an episode of psychosis).

**Methods and design:**

This is an experimentally designed, multi-center randomized controlled trial, with a 3-month follow-up period. The study participants will be 48 patients diagnosed with schizophrenia (first episode) and 48 with a high-risk mental state, from Santiago, Chile, aged between 15 and 35 years. Participants will be submitted to a mindfulness-based intervention (MBI), which will involve taking part in eight mindfulness workshops adapted for people with psychosis. Workshops will last approximately 1.5 hours and take place once a week, over 8 weeks. The primary outcome will be the cognitive function through Measurement and Treatment Research to Improve Cognition in Schizophrenia (MATRICS) and the secondary outcome will be psychological well-being measured by self-reporting questionnaires.

**Discussion:**

The outcomes of this trial will add empirical evidence to the benefits and feasibility of MBIs for the psychotherapeutic treatment of patients with schizophrenia and high-risk mental states in reducing cognitive impairment in attention, working memory, and social cognition, as well as increasing the psychological well-being by empowering the patients’ personal resources in the management of their own symptoms and psychotic experiences.

**Trial registration:**

ISRCTN registration number ISRCTN24327446. Registered on 12 September 2016.

**Electronic supplementary material:**

The online version of this article (doi:10.1186/s13063-017-1967-7) contains supplementary material, which is available to authorized users.

## Background

According to projections of the World Health Organization, 15% of all human disabilities will be associated with mental illnesses by 2020 [1]. One of the mental disorders with the largest social impacts due to high personal and family costs is psychosis, particularly schizophrenia [2]. At present, national and international clinical guidelines suggest favoring an intervention approach that aims to minimize the time an individual is without treatment and to continue treatment during all the phases of the illness. This has led several countries to modify their public policies towards an early intervention of the disorder. Evidence suggests a better prognosis for patients with a lower duration of untreated psychosis [3, 4].

Early intervention not only focuses on the first psychotic episode but also in premorbid states, characterized by subclinical signs and symptoms and with specific features, including delusional beliefs, attenuated hallucinations, and disorganized speech with intact reality testing but of sufficient severity and/or frequency that it is not discounted or ignored. Additionally, symptoms should have worsened in the past year with sufficient clinical discomfort for the affected patients or their relatives to pursue specialized help. Such attenuated symptoms have been characterized as a high-risk mental state for psychosis, collected by DSM-5 to be studied as an Attenuated Psychosis Symptom Syndrome [5]. While not all individuals who experience a high-risk mental state derive into a psychosis, all those with schizophrenia showed a high-risk premorbid state. Therefore, it is extremely relevant to determine the factors that effectively predict a transition towards psychosis [6].

Several countries, including the United Kingdom, Australia, or Canada, to name a few, have included early intervention as a core approach to address schizophrenia. In Chile, its treatment has been a public policy priority. As a matter of fact, first-episode schizophrenia is the first mental health condition included in the Explicit Health Care Guarantees. Clinical guidelines suggest an integral approach, i.e., one that considers the psychosocial and pharmacological dimensions. Among the psychosocial interventions with the best results in addressing schizophrenia and other psychotic disorders at the world level is cognitive behavioral therapy. Recently, cognitive behavioral therapy has introduced several tools and strategies that promote psychological processes based on acceptance and mindfulness [7].

### Mindfulness-based interventions (MBIs)

Mindfulness is a research area in psychology that has experienced the greatest development in recent years. These interventions include acceptance and commitment therapy (ACT), mindfulness-based cognitive therapy, mindfulness-based stress reduction therapy, or dialectical behavior therapy. This set of procedures shares the basic notion that, in order to achieve adequate psychological functioning, individuals should change the way in which they relate to their symptoms instead of eliminating them [8]. Recently, these interventions have been grouped under the label of third wave cognitive behavioral therapies [9]. ACT and MBIs are among the third wave therapies used efficiently in psychosis.

Mindfulness can be understood as a specific form of meditation that seeks to increase different psychological functions by means of a synergic effort between attention regulation, self-awareness and emotional regulation, thereby increasing psychological resilience and self-regulation [10]. Mindfulness has been defined as, “*Paying attention in a particular way: on purpose, in the present moment, and non-judgmentally*” [11]. It has also been conceptualized as a theoretical construction, as a practice and as a psychological process [12]. Training in mindfulness from a Western and academic standpoint has been developed and disseminated through standardized group programs lasting from 8 to 10 weeks and in clinical and health contexts [8, 13].

Such approaches rely on broad empirical support. In fact, recent meta-analyses suggest that mindfulness is an effective intervention for the treatment of several mental health disorders, including depression and anxiety [14, 15]. However, one recent meta-analysis revealed that ACTs and MBIs show promising results in the management of positive and negative symptoms of psychosis [16].

Specifically, the first study of mindfulness in psychosis was conducted in the United Kingdom with patients who suffered stress-induced hallucinations. Patients attended to six mindfulness sessions. The outcomes of this non-controlled study revealed an increase of mindfulness skills in the management of stress-causing thoughts and images. Additionally, the perception regarding the psychological well-being of patients had improved [17]. On the other hand, Chadwick et al. [17] suggested a series of changes to the MBIs when used in psychotic patients in order to ensure the procedure’s feasibility in this population group. Subsequently, Chadwick et al. [18] performed the first controlled study assessing mindfulness skills and overall patient functioning. Both measures improved in the expected direction and showed significant outcomes in intra-group comparisons, but did not do better than the control group. The second controlled clinical trial was conducted in Spain with a small sample of psychotic patients [19], and revealed that, when patients increased their acceptance rates, they also had a less stressing relationship with their thoughts and emotions, in addition to improvements in overall functioning; however, only the first outcome was statistically significant compared to the control group. Another more solid study in terms of the number of participants (N = 98) and the introduction of follow-up measures (18 months) was performed by Lee and Chien [20]. The outcomes of their study revealed that patients showed improved levels of illness insights and overall functioning, and a reduction in symptom severity and in the number and length of hospitalizations. All of these indicators were statistically significant compared to the control group (usual treatment). Recent studies have confirmed the results mentioned above [21, 22]. Other non-controlled studies have proven the feasibility of using mindfulness in patients with psychosis [23–25].

On the other hand, several qualitative studies have revealed that practicing mindfulness results in a less stressful relationship between patients and their symptoms, with greater flexibility and control over them. This change has a direct impact on the way in which a person relates both to others and to themselves, increasing their confidence and the sense of self-agency. In other words, a person gains an active role in the way they face their symptoms and life, strengthening a self-image that is not only centered around the absence or presence of psychotic symptoms but on their personal resources as the main focus of recovery [26–28].

Considering all the research in this area, i.e., more than 20 studies including randomized, non-controlled, qualitative, and single case clinical trials [29], two aspects can be pinpointed concerning the use of mindfulness in psychosis. Firstly, that mindfulness reduces the stress associated to psychotic symptoms and thereby promotes patient recovery and, secondly, its implementation in clinical centers is both safe and viable.

### The relevance of the mindfulness study in cognitive functions and psychological well-being

#### Mindfulness and cognitive functions

Cognitive impairments have been described as a core element of schizophrenia; indeed, 90% of patients have difficulties in this area [30, 31]. What is relevant is that these impairments are found before the onset of schizophrenia, which suggests that they are not a consequence of the illness but rather a vulnerability factor for schizophrenia [32] and a functionality predictor in patients [33]. Cognitive functions subject to the greatest decline in the transition from a high-risk mental state to a first psychotic episode are working memory [34], attention [35], and social cognition [36]. For instance, working memory has been defined as the most solid indicator in the transition between a high-risk mental state and schizophrenia. On the other hand, improvements in attention could be a remission indicator in high-risk people with schizophrenia [35], while the reduction of social cognition skills have been related to the length and severity of symptoms [37].

The control of attention in mindfulness is one of the pillars to achieve improved psychological flexibility and therefore greater response options facing adverse stimuli. In fact, expert meditators score higher in attention tests than non-meditators or beginners [38]. On the other hand, working memory has been related to other cognitive domains that are impaired in schizophrenia, such as attention and planning [39], and other executive functions, including inhibitory control and mental flexibility [40]. As background information, while mindfulness has a major impact on working memory [41, 42], this impact depends on the time devoted to meditation, being greater in people who meditate regularly compared to beginners [42, 43]. Similarly, working memory has been related to social cognition [44]. For instance, the cognitive training of working memory improves social perception in patients with schizophrenia [45].

#### Mindfulness and its impact on psychological well-being

Psychological well-being is a key area in the current conception of health [1]. In other words, it is not only the absence or reduction of symptoms that matters in the treatment of severe mental disorders such as schizophrenia [46]. The concept of mental health recovery notes that people should lead a purposeful and meaningful life, regardless of whether they have a mental disorder. However, a review study concluded that only a very small amount of interventions have proven effective in improving the psychological well-being of patients with schizophrenia [47]. Numerous studies, however, emphasize the favorable impacts of mindfulness, among them a reduction of symptoms and, above all, the development of a more favorable way of relating to own experience, with greater presence and acceptance, thereby promoting the sense of self-empowerment and improving a broader self-image not exclusively focused on the presence or absence of symptoms [21, 28]. In this sense, focusing attention on own feelings and managing them is related to improved emotional intelligence, also a very important aspect in the social sphere [48, 49]. An increase in the aspects mentioned above is among the main findings of qualitative research concerning mindfulness in psychosis [31]. It should be noted that this project understands psychological well-being from two perspectives. The first refers to a positive dimension which aims at increasing positive emotions and more flexible psychological states (mindfulness), while the second refers to a symptomatic sphere where a less aversive relationship with private events is expected and thus a reduction in psychological discomfort [46].

The purpose of this study is to determine how efficient MBIs can be in increasing cognitive functions and psychological well-being in patients with a first episode of schizophrenia and patients in a high-risk mental state, and thus define the scope of mindfulness to increase these areas of functioning. The specific goals are to (1) determine how efficient a mindfulness intervention can be in increasing attention, working memory, and social cognition rates, compared with the control group; (2) establish which spheres of psychological well-being increase (positive dimension) and which decrease (symptomatic dimension) with mindfulness training, compared to the control group; (3) determine whether the expected outcomes (increased cognitive functions and psychological well-being) are still present 3 months after the intervention has finalized, and additionally establish whether impacts are greater in participants who continue practicing mindfulness compared to those who fail to continue the practice; and (4) describe, on the one side, the subjective experience of patients regarding the feasibility of implementing such an intervention in a local context and, on the other, the benefits of mindfulness for patients compared to other psychosocial interventions in the context of mental health public policies in Chile.

The hypotheses are as follows. (1) MBIs will increase executive functions. Specifically, a statistically significant increase is expected in attention, working memory, and social cognition rates compared to the control group. (2) Subjective psychological well-being rates will increase significantly in the mindfulness group compared to the control group. (3) The outcomes in the areas described will persist 3 months after; however, impacts will be greater on people who continue practicing mindfulness compared to those who do not.

The questions that guide the qualitative goals are as follows. (1) What were the benefits and shortcomings found during the workshop? (2) Do patients continue with this practice and mainstream the outcomes into other areas of their lives? (3) What differences and similarities are perceived by patients between mindfulness and traditional psychosocial interventions?

## Methods and design

This is a multi-center randomized controlled trial (RCT) with two parallel arms and 1:1 allocation. A mixed analysis approach will be used. To assess the RCT’s effectiveness, a quantitative method will be used, while a qualitative approach will help understand subjective change processes considering cultural traits.

### Participants

**Fig. 1:**
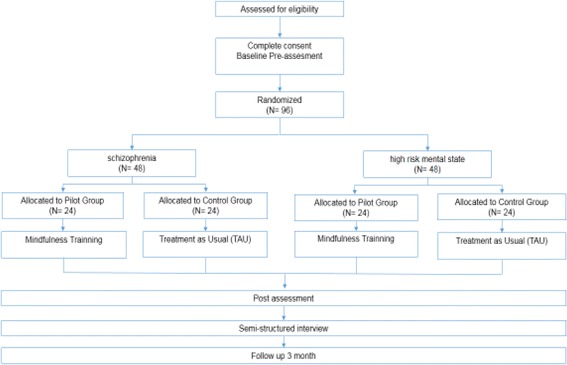
This figure illustrates the process of randomization, allocation assessment, and follow-up throughout the study

**Fig. 2:**
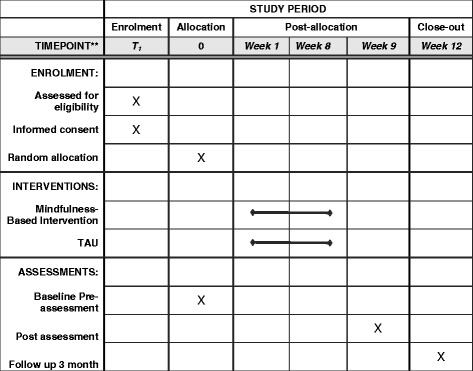
Schedule of enrollment, intervention, and assessments for the mindfulness-based intervention

Patients will be recruited from six clinical centers from three different cities in Chile; four in Santiago, one in Valdivia, and one in Osorno. Ninety six people are expected to take part in the study, of which 48 will be patients diagnosed with a first episode of schizophrenia (divided into 24 trial and 24 controls) based on the following criteria: patients with a psychotic episode without a prior diagnostic of schizophrenia, patients diagnosed with schizophrenia for the first time, and/or patients who persist with the schizophrenia diagnosis after the first episode. Additionally considered is the participation of 48 patients under a high-risk mental state (divided into 24 trial and 24 controls) who meet the criteria of an Attenuated Psychosis Symptom Syndrome, including delusional beliefs, attenuated hallucinations, and disorganized speech with intact reality testing but of sufficient severity and/or frequency that it is not discounted or ignored [5]. Sample size was estimated considering a previous RCT for people with schizophrenia on both the MBI [20] and a cognitive remediation program [50]. The sample size was calculated by using the statistical program G*Power [51]. The sample required to detect statistically significant differences in the cognitive functioning for a two-tailed test of the proportions with an effect size of 0.80, an alpha of 0.05, and a power of 0.90 is 2 × 39. With an estimated 15–20% drop-out over 3 months, we decided to include 96 persons in the trial, 48 allocated in each intervention arm. The inclusion criteria are (1) patients diagnosed with a first episode of schizophrenia or in high-risk of psychosis, as applicable; (2) aged between 15 and 35 years; and (3) clinical stability defined by medical and psychometric criteria (e.g., PAANS). Exclusion criteria are (1) risk of suicide; (2) severe intellectual disability (mental retardation); (3) medical illness inconsistent with the intervention; and (4) substance abuse or dependence in the past 6 months. Figures 1 and 2 summarize the process of randomization, group allocation, and assessment. The SPIRIT checklist is available in Additional file 1.

### Procedure

Participants, as defined above, will be randomly allocated (a simple randomized sampling will be used) to the trial or control group. Randomization to either trial or treatment as usual will be conducted by independent researchers using a stratified block randomization procedure with a computer-generated allocation sequence. Health experts who will implement the MBIs will be blind to this procedure and will not carry out the assessment.

#### Intervention group

Participants will be provided with the MBI, plus treatment as usual, divided into 24 patients diagnosed with a first episode of schizophrenia and 24 patients in a high-risk mental state (groups do not include both profiles). This means that both groups will be submitted to eight sessions of mindfulness workshops adapted for patients with psychosis [17]. The proposed duration for each session is 1 hour and a half, once a week. The intervention will take place in the facilities of each participating clinical center and led by a mindfulness coach with specific training in the target audience. The main project researcher will supervise and train each coach in the application of mindfulness in psychosis. The workshop includes take-home exercises. Additionally, every participant will get a pen drive or CD with guided mindfulness practice audios and a booklet with the contents of each session. Groups will be composed of up to eight members and two workshops have been planned by every participating institution.

#### Control group

Participants will receive standard care for this illness (treatment as usual), in other words, pharmacology and psychosocial intervention under clinical guidelines (e.g., social skills workshop).

The assessment, application of neuropsychological tests (Measurement and Treatment Research to Improve Cognition in Schizophrenia (MATRICS) Battery), and self-reporting questionnaires will take place (1) before the workshop; (2) after the workshop; and (3) at follow-up 3 months after completing the workshop. Additionally, 4 weeks after completing the workshop, participants will be submitted to semi-structured interviews. The number of interviews will be defined based on the saturation of the concepts found in the qualitative methodology. Each interview will last approximately 20 minutes and will be performed by a member of the research team.

### Measuring instruments

#### Primary outcome

The primary outcome will be the cognitive function through MATRICS. This battery assesses seven functioning areas, namely speed of processing, attention/vigilance, working memory, verbal learning, visual learning, reasoning and problem solving, and social cognition. The assessment takes place through seven tests.

#### Secondary outcomes

##### Psychological well-being/positive dimension (increase)

The following instruments will be used to assess the psychological well-being in its positive dimension:

1. Psychological well-being scale [52, 53], which includes 39 items grouped in six subscales: self-acceptance, positive relations with others, autonomy, environmental mastery, purpose in life, and personal growth. Respondents answer in the Likert scale format with a range from 1 to 6 (1 = strong disagreement and 6 = strong agreement). This scale has been broadly studied to assess psychological well-being. The version validated in Chile and published by Véliz-Burgos will be used [54].
2. Self-esteem scale by Rosenberg. This is a 10-item scale broadly used in research. It is a uni-dimensional instrument answered with a Likert scale of four options, ranging from “strongly agree” to “strongly disagree”. The Chilean version showed adequate psychometric properties [55].
3. Five Facet Mindfulness Questionnaire [56], which is a self-reporting questionnaire that describes mindfulness operationally as a multidimensional construction, built on the following five facets: observing, describing, acting with awareness, non-judging of experiences, and non-reactivity to experience. The questionnaire uses a Likert scale ranging from 1 (never or very rarely true) to 5 (very often or always true). It presents adequate psychometric properties in the Chilean population [57].

##### Psychological well-being/symptomatology dimension (reduction)

The following instruments will be used to measure the symptomatic dimensions of psychological well-being:

1. Positive and Negative Affect Schedule (PANAS) [58], which is a 20-item scale including by 10 items that assess positive affect and 10 items that assess negative affect. Items are formed by words that describe different feelings and emotions. The Chilean version published by Dufey and Fernández will be used [59].
2. Penn State Worry Questionnaire (PSWQ-11) [60], which is a measure of worry phenomena designed to assess the general trends when experiencing worry. It is composed by 16 items to which participants respond in a 5-score Likert scale, ranging from 1 “not at all typical of me” to 5 “very typical of me”. The Spanish version of Sandin, Chorot, Valiente and Lostao will be used, which has shown to have adequate psychometric properties [61].
3. Depression, Anxiety and Stress Scale (DASS-21) [62]. This is a self-applied scale that assesses general symptoms in three dimensions. Seven items are related to depression symptoms, seven to anxiety symptoms, and seven to stress. The scale has been validated in Chile with good psychometric properties [63].

### Data analysis

A mixed qualitative and quantitative analysis method will be used. Qualitative information will be collected by means of semi-structured interviews and assessed with the Grounded Theory. This data analysis approach is widely used in social sciences and was previously applied for the analysis of the subjective experience of patients with psychosis [26]. This methodology will analyze the patients’ subjective experience regarding the feasibility and contribution of this type of intervention in a local context. At the quantitative level, the hypothesis will be contrasted though a mixed designed ANOVA in order to consider both the impacts of the intervention (inter subjects) and time (intra-subject; pre, post, and follow-up measurements) in every variable assessed. The analysis will help prove the hypotheses regarding the efficiency of this intervention in both patient groups. If statistically significant differences are found between the trial and control groups at the beginning of the intervention, corrections will be conducted using an ANCOVA.

### Ethics

The research protocol follows the indications of the Singapore Statement on Research Integrity and has been approved by two ethics committees, namely Pontificia Universidad Católica de Chile and Universidad de Chile.

## Discussion

Schizophrenia is a chronic and severe mental health disorder. In Chile, it is a public health priority subject to integral treatment indications (pharmacological and psychosocial). There is abundant empirical evidence suggesting that MBIs are feasible and beneficial interventions for patients with schizophrenia. MBIs promote a more flexible relationship with the psychotic symptoms, with impacts on the patients self-awareness and own resources and not only on the shortcomings and limitations of having a chronic mental illness [7, 16, 29]. However, one of the elusive areas in targeting these psychotherapeutic treatments are cognitive impairments [7], while an adequate cognitive functioning has clear impacts on the psychological well-being of patients [64].

All the scientific evidence points to the need to study the relationship suggested in this project, i.e., to determine the impact of mindfulness on cognitive functions and the psychological well-being in people with schizophrenia. However, it is also worthwhile to include people with a high-risk mental state since this could provide insights in several areas, including (1) proving the efficacy of the MBI intervention for this group; (2) comparing the outcomes with those of patients with a first episode of schizophrenia (i.e., determine the scope of mindfulness according to the pathology level); and (3) allow subjective assessment of the mindfulness benefits according to patient experience when facing a reduction in psychological discomfort.

Thus, this study aims, on the one hand, to discuss different aspects that have not yet been addressed in the literature concerning MBIs applied in psychosis and, on the other, to confirm previous findings in other social and cultural contexts so as to improve the ecological soundness of this intervention [28]. In addition, the insights provided by self-reporting studies need to be improved through more accurate measurements such as neuropsychological tests.

Should the hypotheses suggested in this study be proven, this would add empirical evidence about the benefits and feasibility of MBIs for the psychotherapeutic treatment of patients with schizophrenia as well as of those with a high-risk mental state in reducing cognitive impairments in attention, working memory, and social cognition. The results would also increase the psychological well-being by empowering patients’ personal resources in the management of their own symptoms and psychotic experiences.

### Trial status

The research is presently in the sample recruitment phase and the workshops are expected to begin on November 15, 2016.

## Additional files

Additional file 1: SPIRIT checklist. (DOC 121 kb)

## Abbreviations

ACT: Acceptance and commitment therapy
MATRICS: Measurement and Treatment Research to Improve Cognition in Schizophrenia
MBI: Mindfulness-based intervention
RCT: Randomized controlled trial
